# Supplementary material for: Burrowing detritivores regulate nutrient cycling in a desert ecosystem
Source: Proc Biol Sci. 2019 Oct 30;286(1914):20191647. doi: 10.1098/rspb.2019.1647 (PMC6842856; doi:10.1098/rspb.2019.1647)
Supplement: Additional information regarding climate conditions at the field site (S1), field and lab methods (S2), and statistical analysis (S3) [file rspb20191647supp1.pdf]

Electronic Supplementary Material

Burrowing detritivores regulate nutrient cycling in a desert ecosystem

Nevo Sagi, José M. Grünzweig, Dror Hawlena

Proceedings of the Royal Society B

DOI: 10.1098/rspb.2019.1647

Appendix S1 - Climate conditions at the research site

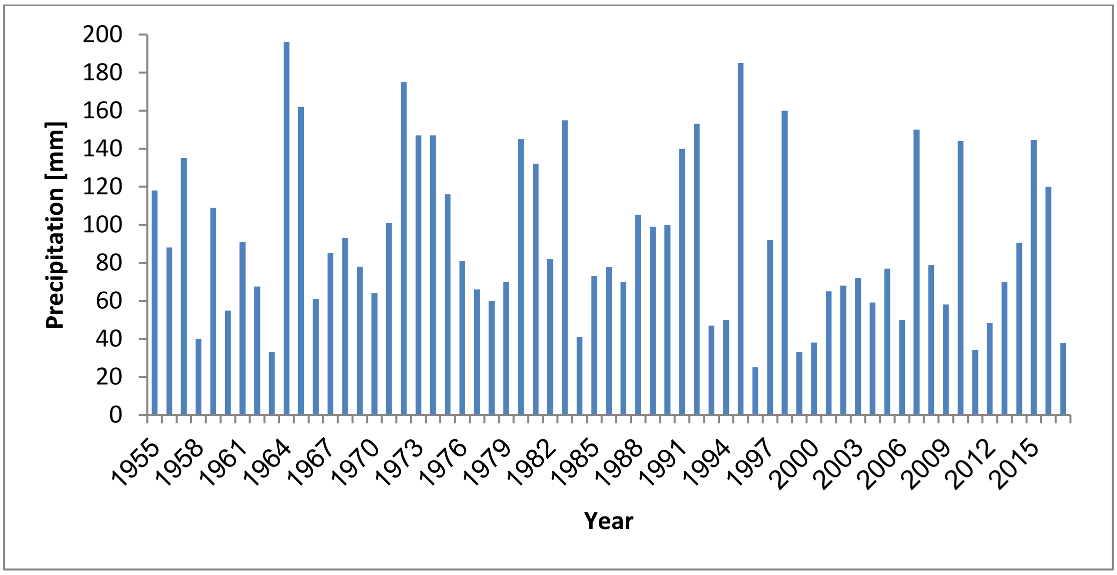

Figure S1-1. Annual precipitation in Even-Ari research station (1955-2017)[1]. Mean annual precipitation is 92.19 mm**,** and the mean absolute difference between two consecutive years is 45.43 mm.

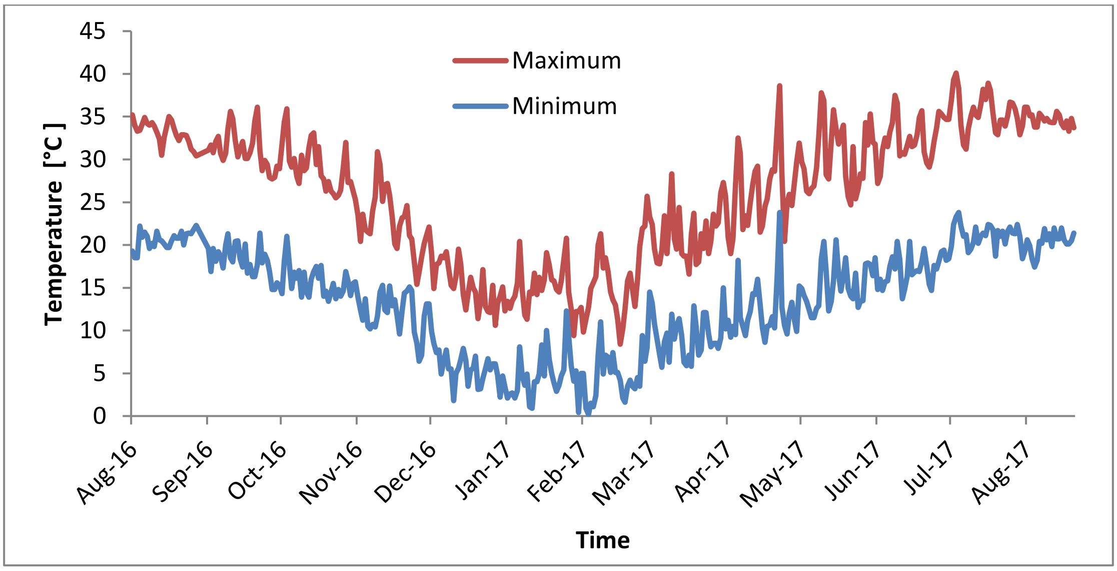

Figure S1-2. Maximum (red) and minimum (blue) air temperatures in Even-Ari research station during the litter baskets experiment[1]. Mean daily maximum and minimum temperatures are 26.1°C and 13.4°C, respectively. Mean daily temperature amplitude is 12.7°C.

Possible association between wind characteristics and litter mass loss

Wind data at our field site was collected in a temporal resolution of 10 minutes, during the entire experimental period[1]. We used this information to explore whether accidental litter loss by wind may contribute to the elevated rates of litter mass loss from the macro-baskets. The data clearly show that average and maximal wind speeds or the number of extreme wind events (above 10 m/s) cannot explain our findings (Table S1-1; Fig. S1-3). For example, both the maximal wind speed and the number and magnitude of strong gusts (above 10 m/s) were highest during the winter when litter mass loss in the macro-baskets was the lowest. The pattern was opposite during the late summer in which we observed the largest rates of litter mass loss, but the average and maximal wind speeds were the lowest and there were no extreme wind gusts that exceed 10 m/s. Thus, even if wind can cause litter loss from our baskets, then the effect size during the summer should be smaller than the one observed during the winter (when winds are strongest) and not much larger as clearly shown by our results.

Table S1-1. Wind speed statistics during the four trials of litter baskets experiment.

Season	Average Wind Speed ± SE [m/s]	Maximal Wind Speed [m/s]	# Extreme Wind Events (above 10 m/s)
Late Summer	3.32 ± 0.02	9.5	0
Winter	3.55 ± 0.03	17.9	382
Spring	3.67 ± 0.02	14.6	110
Early Summer	3.49 ± 0.02	10.2	2

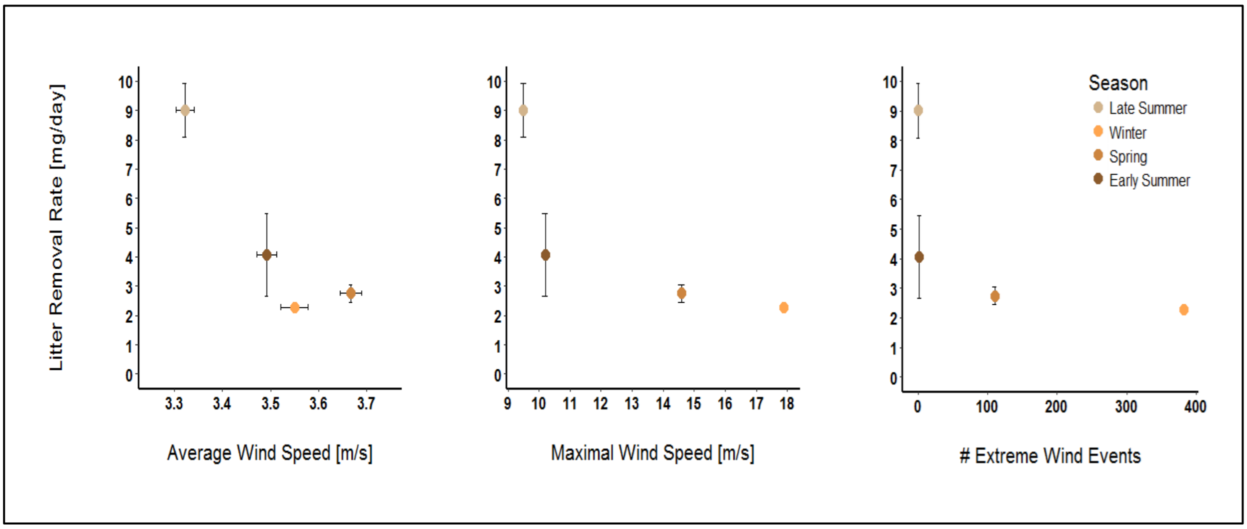

Figure S1-3. Relationship between wind speed and litter removal rate (mean±SE) from macro-baskets during the 4 trials of litter baskets experiment.

Appendix S2 – Materials and Methods – additional information

The contribution of macro-detritivores to surface litter removal

We used square plastic baskets with the following dimensions:

Bottom – 120 X 130 mm

Top – 95 X 105 mm

Height – 55 mm

Hole-Size – 6.5 X 7 mm

Meso and macro-baskets were floored with 200 µm mesh size net (micro-net) in order to avoid litter (2mm<length; thickness~3mm; Fig. S2-1) loss from the bottom while allowing access of micro-organisms. In micro-baskets the litter was placed inside micro-net bags (120 X 130 mm) that served also as the basket floor. After insertion of litter, all baskets were wrapped with 2 mm mesh size aluminum net (meso-net) that prevents termite entry. Macro-baskets were identical to meso-baskets except of 4 openings (10cm X 1cm) that were cut in the meso-net about 1 cm above ground level. This opening allow access of macrofauna while avoiding litter spill from the sides (see Fig. S2-2 for illustration).

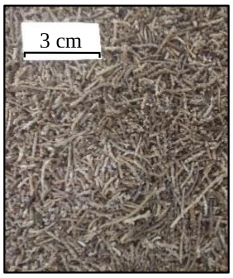

Figure S2-1 – Haloxylon scoparium litter used in the litter baskets experiment.

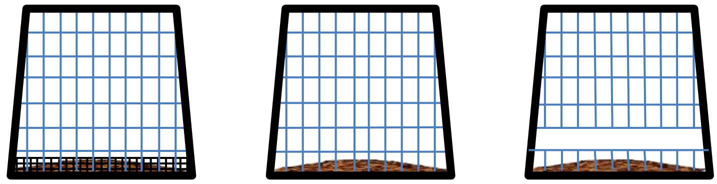

Figure S2-2 - An illustration of the litter baskets design

We monitored all baskets every two weeks to check for litter accumulation near the outer meso-nets, prevent litter spills (from the macro-baskets) and test the effectiveness of our design (observing macro-arthropods behavior). We have never seen accumulation let alone spill, suggesting the 2mm mesh of the litter-baskets

serves as a wind breaker and that accidental litter loss by wind, if at all exist, was minimal. Isopods were observed consuming litter within the baskets.

We transported each pre-weighed basket to the field in a new individual Ziploc bag that was placed within a sturdy container. After positioning the basket in a pre-assigned location, we checked the Ziploc bags for remaining litter and added the leftover litter, if found, to the corresponding basket. After returning to the lab, we rechecked the empty Ziploc bags above a tray covered with white paper and subtracted the weight of the tiny litter remains (rarely found) from the initial litter weight.

At the end of each trial, we carefully removed the arched metal stakes that tightly anchored the baskets to the soil and carefully inserted the baskets into new Ziploc bags for transportation to the lab. In the lab, we removed the baskets and all the litter from the bags above a tray covered with white paper in order to minimize accidental losses. We collected the small litter fragments manually with the aid of a large magnifying glass. In six cases (1 macro, 1 micro and 4 meso baskets) during the first trial, litter was accidently spilled out of the tray. As a precaution, we excluded these measurements from the analysis. We also excluded one meso-basket during the second trial because of possible mass loss that happened while positioning the baskets in the field.

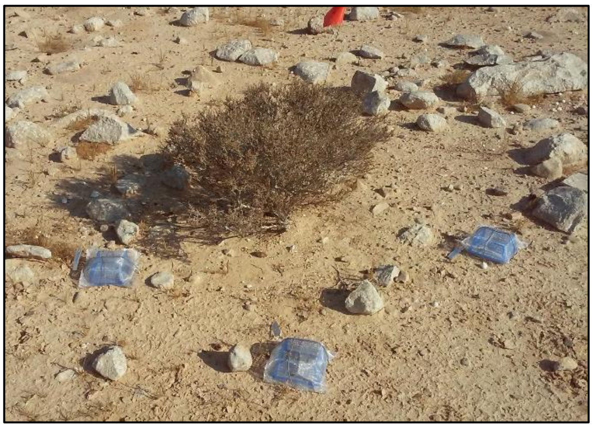

Figure S2-3 – The litter baskets as placed in the field site

Soil characteristics around *Hemilepistus reaumuri* burrows

We first sampled the feces mounds around isopod burrows, as well as the soil crust at a distance of 5 cm from the outer boundaries of the mound (figure S2-4).

After removing the mound and the soil crust, we took 2 cm diameter samples of the upper 30 cm of the soil profile, at different distances (0, 10, and 20 cm) from the burrow opening (figure S2-5).

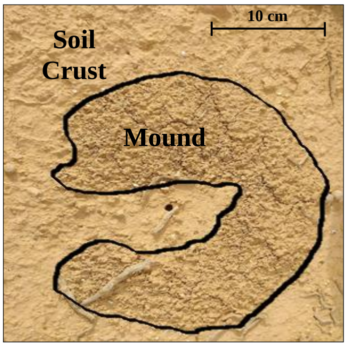

Figure S2-4 – An isopod burrow before sampling. Mound and soil crust are marked. Dark spot at the center is the burrow opening.

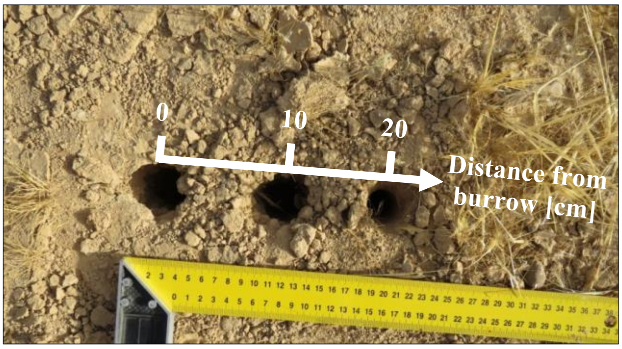

Figure S2-5 – Top view of the soil adjacent to an isopod burrow, after soil sampling.

Microcosms experiment (Isopolis)

We constructed five custom-made Plexiglas microcosms that allow detailed monitoring of isopod activity and nutrient distribution belowground. Isopolis was made of an aboveground chamber (dimensions 60*30*20cm) connected to a narrow (60*40*0.8cm) belowground transparent chamber (Fig. S2-6A). We filled the aboveground chamber with 5 cm deep homogenized field collected soil and paved it with field collected BSC. The belowground chamber was compacted with the same homogenized soil, to which we translocated one *H. reaumuri* mating pair (Fig. S2-6B). The transparent belowground chambers were covered by opaque screens (Fig. S2-7A). Twice a week throughout the experiment we temporarily removed the opaque screens to record the distribution of isopods and feces within the burrows (Fig. S2-7B). We used this information to determine the isopods and feces frequencies of occurrence in different depths. At the end of experiment, we carefully removed the front wall of the belowground chamber and sampled the soil at 1 cm intervals from the burrow wall to a distance of 10 cm. We repeated this sampling protocol from the surface to the burrow maximal depth in 5 cm intervals (Table S2-1).

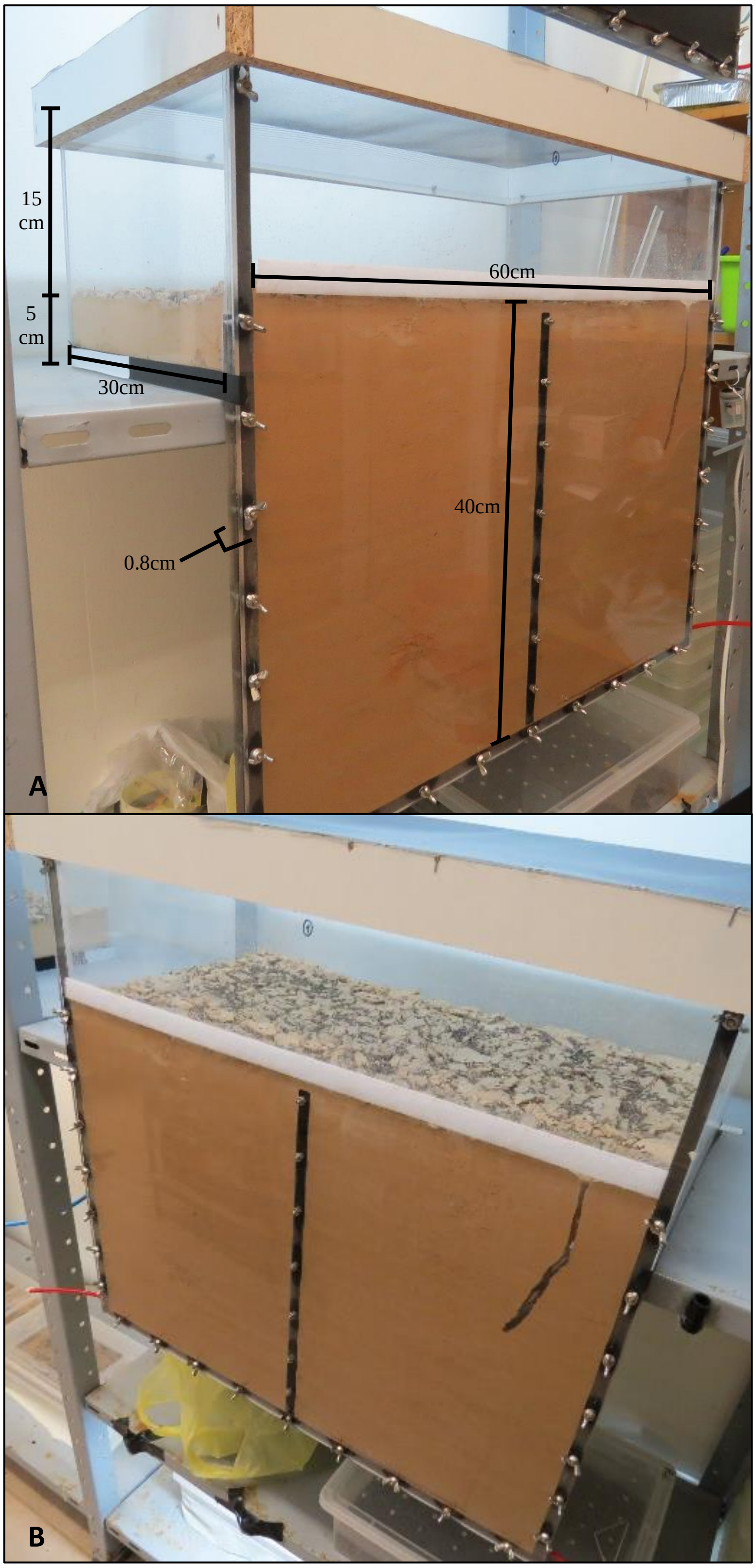

Figure S2-*6* – An informative view of Isopolis, exhibiting the dimensions and soil depth of the above- and belowground chambers (A), the surface-covering biological soil crust with *Haloxylon scoparium* litter scattered above, and a pair of isopods inside the burrow (B). The horizontal white stripe is a Velcro fastener for connecting the opaque front screen.

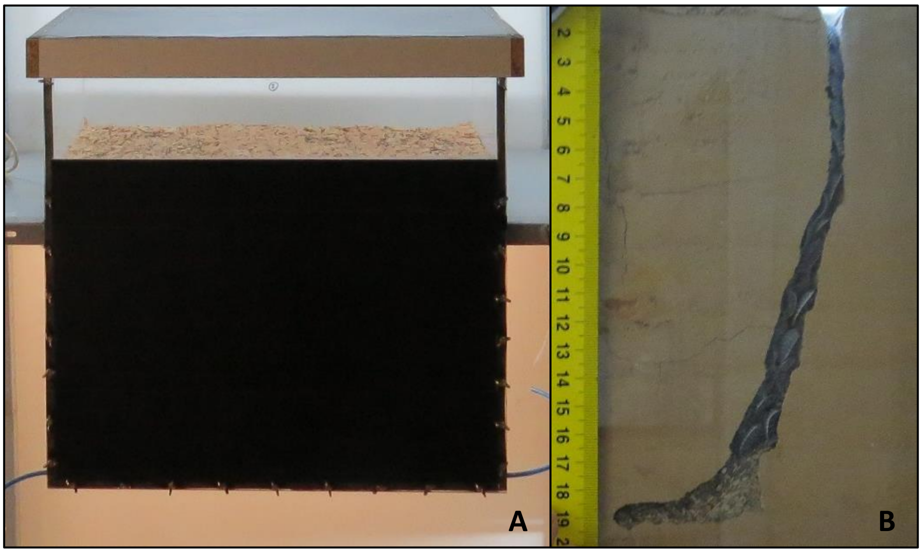

Figure S2-7 – (A) A front view of Isopolis with the opaque front screen in place. (B) A photo of an Isopolis burrow, used for calculation of isopods and feces frequencies of occurrence in different depths. In this case feces occur in the deepest 2 cm of the burrow, while isopods occupy the rest of it except the uppermost 1 cm.

Table S2-1 – Maximal and sampled depths in each microcosm.

Isopolis	Burrow Maximum Depth [cm]	Sampled Depths [cm]
1	14	0-1, 4-5, 9-10, 13-14
2	20	0-1, 4-5, 9-10, 14-15, 19-20
3	19	0-1, 4-5, 9-10, 14-15, 18-19
4	19	0-1, 4-5, 9-10, 14-15, 18-19
5	14	0-1, 4-5, 9-10, 13-14

Appendix S3 – Statistical analysis – additional information

All analyses were done using RStudio software [2], 1.1.447 version. Linear mixed models (LMMs) were fitted and Likelihood ratio tests (LRTs) were done using R package 'lme4' [3]. Paired Student's t-tests were done using R package 'stats' [4]. Post hoc comparisons were done using R package 'lsmeans' [5]. Plots were generated using R package 'ggplot2' [6].

The contribution of macro-detritivores to surface litter removal

We used mean-centered dummy coding for contrasts of both basket type and trial. This definition of contrasts allowed us to compare between levels of the factors, while avoiding artificial collinearity between the two fixed effects. The models that were used in this analysis are depicted in table S3-1.

We used a series of LMMs to analyze whether the rates of litter removal differ between the three basket types in each trial separately. We divided the data to four subsets, one for each trial. For each subset we ran an LMM with basket type as fixed effect and with by-bush random intercept. We then tested the significance of the fixed effect using LRT, comparing models that include and do not include the fixed effect. The models that were used in this analysis are depicted in table S3-2.

Table S3-1 – Models used for analysis of the effect of basket type on litter removal rate across 4 different trials.

Model	Fixed Effects	Random Effects
Full Litter Model	Basket Type*Trial	By-Bush random slope for Basket Type, By-Bush random intercept
Without Basket Type	Trial	By-Bush random slope for Basket Type, By-Bush random intercept
Without Trial	Basket Type	By-Bush random slope for Basket Type, By-Bush random intercept
Without Interaction	Basket Type+Trial	By-Bush random slope for Basket Type, By-Bush random intercept

Table S3-2 - Models used for analysis of the effect of basket type on litter removal rate in each trial separately.

Model	Fixed Effects	Random Effects	Data Subset
Late Summer	Basket Type	By-Bush random intercept	Late Summer
Late Summer – No Basket Type	None	By-Bush random intercept	Late Summer
Winter	Basket Type	By-Bush random intercept	Winter
Winter – No Basket Type	None	By-Bush random intercept	Winter
Spring	Basket Type	By-Bush random intercept	Spring
Spring – No Basket Type	None	By-Bush random intercept	Spring
Early Summer	Basket Type	By-Bush random intercept	Early Summer
Early Summer – No Basket Type	None	By-Bush random intercept	Early Summer

Inclusion of by-bush random slopes in these models was not applicable using the 'lme4' package, due to over-parameterization. We performed an additional analysis incorporating by-bush random slopes using R package 'nlme' [7]. The results of this analysis are depicted in table S3-3.

Table S3-3 – Results of statistical analysis using R package 'nlme': (A) Likelihood ratio tests (LRTs) testing the effects of litter basket type, trial and their interaction on litter removal rates. (B) LRTs testing the effect of litter basket type on litter removal rates in each trial separately. (C) Pairwise comparisons between litter removal rates in different litter basket types.

A. *LRTs for all data* [1]					
**Effect**		**dAIC**	**L**	**df**	**P-value**
Litter Basket Type		128.3	144.3	19,11	<0.0001
Trial		103.8	121.8	19,10	<0.0001
Interaction		92.1	104.1	19,13	<0.0001
B. *LRTs for each trial separately* [II]					
**Trial**		**dAIC**	**L**	**n**	**P-value**
Late Summer		39.31	45.31	84	<0.0001
Winter		105.76	111.76	89	<0.0001
Spring		89.09	95.09	90	<0.0001
Early Summer		34.66	40.66	90	<0.0001
C. *Post Hoc comparisons (Tukey)*					
**Trial**	**Basket Types Compared**	**Difference (mg/day)**	**df**	**t**	**P-value**
Late Summer	Macro-Micro	9.04 ±0.91	52	9.97	<0.0001
	Macro-Meso	9.51±0.96	52	9.93	<0.0001
	Meso-Micro	-0.47±0.22	52	-2.15	0.09
Winter	Macro-Micro	1.06±0.13	57	7.99	<0.0001
	Macro-Meso	0.32±0.10	57	3.21	<0.01
	Meso-Micro	0.74±0.11	57	7.05	<0.0001
Spring	Macro-Micro	2.31±0.32	58	7.33	<0.0001
	Macro-Meso	2.26±0.32	58	7.13	<0.0001
	Meso-Micro	0.05±0.04	58	1.28	0.41
Early Summer	Macro-Micro	3.69±1.42	58	2.80	<0.05
	Macro-Meso	3.96±1.41	58	2.59	<0.05
	Meso-Micro	-0.27±0.06	58	-4.37	<0.001

I. n=353.

II. df = 10,7 in all trials.

Table S3-4 - Results of Likelihood ratio tests for the effects of litter basket type, trial and their interaction on litter removal rates, with by-bush random intercept, by-bush random slope for basket type, and by-initial mass random intercept (n=353).

**Effect**	**dAIC**	**χ^2**	**df**	**P-value**
Litter Basket Type	191	216.96	13	<0.0001
Trial	101.1	119.1	9	<0.0001
Interaction	92.1	104.06	6	<0.0001

Soil characteristics around *Hemilepistus reaumuri*'s burrows

Data of moisture content and electrical conductivity (EC) in mounds and soil crust were log-transformed, in order to meet the assumptions of paired t-tests. Data of moisture and NO3 content in soil were log-transformed in order to meet the assumptions of LMM. One measurement of soil PO4 content was marked as an outlier and was removed from data before analysis.

Table S3-5 – Models used for analysis of the effect of distance from burrow on different soil properties

Soil Property	Response Variable	Fixed Effects	Random Effects
PO4	PO4 concentration [μg g^{-1}]	Distance from Burrow	By-Burrow random intercept
NO3	Log (1+NO3 concentration [μg g^{-1}])	Distance from Burrow	By-Burrow random intercept
NH4	NO4 concentration [μg g^{-1}]	Distance from Burrow	By-Burrow random intercept
Microbial Biomass	Substrate-Induced Respiration [μg C-CO_2 g $soil^{-1}$ h^{-1}]	Distance from Burrow	By-Burrow random intercept
Moisture Content	Log (% Water)	Distance from Burrow	By-Burrow random intercept
Salinity	Electrical Conductivity [mS cm^{-1}]	Distance from Burrow	By-Burrow random intercept
Acidity	pH	Distance from Burrow	By-Burrow random intercept

Total soil N content in lab microcosm experiment (Isopolis)

N content data were log-transformed in order to meet the assumptions of LMM.

Table S3-6 – Models used for analysis of the effect of distance from burrow, isopods and feces frequencies of occurrence on soil total N content.

**Model**	**Fixed Effects**	**Random Effects**
Full Model	Distance from burrow + Isopods FO[1] + Feces FO	By-Isopolis random intercept
Without Distance	Isopods FO + Feces FO	By-Isopolis random intercept
Without Isopods	Distance from burrow + Feces FO	By-Isopolis random intercept
Without Feces	Distance from burrow + Isopods FO	By-Isopolis random intercept

[1] Frequency of Occurrence

References

1. Israel Meteorological Service. 2019 Israel Meteorological Service. See http://www.ims.gov.il/IMSENG/All_Tahazit/homepage.htm (accessed on 9 January 2019).

2. RStudio Team. 2016 RStudio: Integrated Development for R.

3. Bates D, Maechler M, Walker S. 2015 Fitting Linear Mixed-Effects Models Using ‘lme4’. *J. Stat. Softw.* **67**, 1–48. (doi:10.18637/jss.v067.i01)

4. R Core Team. 2018 R: A language and environment for statistical computing. *R Found. Stat. Comput.* (doi:http://www.R-project.org/)

5. Lenth R V. 2016 Least-Squares Means: The ‘R’ Package ‘lsmeans’. *J. Stat. Softw.* **69**, 1–33. (doi:10.18637/jss.v069.i01)

6. Wickham H. 2009 *ggplot2: Elegant Graphics for Data Analysis*. New York: Springer-Verlag.

7. Pinheiro J, Bates D, DebRoy S, Sarkar D. 2018 ‘nlme’: Linear and Nonlinear Mixed Effects Models. See https://cran.r-project.org/package=nlme.
